# MicroRNA-127-5p regulates osteopontin expression and osteopontin-mediated proliferation of human chondrocytes

**DOI:** 10.1038/srep25032

**Published:** 2016-04-29

**Authors:** Min Tu, Yusheng Li, Chao Zeng, Zhenhan Deng, Shuguang Gao, Wenfeng Xiao, Wei Luo, Wei Jiang, Liangjun Li, Guanghua Lei

**Affiliations:** 1 Department of Orthopaedics, Xiangya Hospital, Central South University, Changsha 410078, China; 2 Department of Orthopaedics, Second People’s Hospital of Jingmen, Jingmen, 448000, China; 3 Department of Bone and Joint, Shenzhen People’s Hospital, Second Clinical Medical College of Jinan University, Shenzheng, 518020, China; 4 Department of Joint Surgery, Changsha Central Hospital, Changsha, 410000, China

## Abstract

The aim of this study was to determine the specific microRNA (miRNA) that regulates expression of osteopontin (OPN) in osteoarthritis (OA). The potential regulatory miRNAs for OPN messenger RNA (mRNA) were predicted by miRNA prediction programs. Among eight potential regulatory miRNAs, miR-220b, miR-513a-3p and miR-548n increased, while miR-181a, miR-181b, miR-181c, miR-181d and miR-127-5p decreased in OA patients. miRNA-127-5p mimics suppressed OPN production as well as the activity of a reporter construct containing the 3′-UTR of human OPN mRNA. In addition, mutation of miR-127-5p binding site in the 3′-UTR of OPN mRNA abolished miR-127-5p-mediated repression of reporter activity. Conversely, treatment with miR-127-5p inhibitor increased reporter activity and OPN production. Interestingly, miR-127-5p inhibited proliferation of chondrocytes through OPN. In conclusion, miRNA-127-5p is an important regulator of OPN in human chondrocytes and may contribute to the development of OA.

Osteoarthritis (OA) is regarded as the most prevalent chronic joint disease, and is characterized by a group of mechanical abnormalities, such as degradation of articular cartilage, thickening of subchondral bone, and synovial inflammation^1^,^2^,^3^. There is a growing knowledge and understanding on the pathogenesis of OA^3^. Osteopontin (OPN) is a 44~75 KD multifunctional phosphoprotein, and is associated with the pathogenesis of OA^4^. OPN regulates expression of various factors associating with the pathogenesis of OA, including matrix metalloprotease 13 (MMP13)^5^, hypoxia-inducible factor-2α^6^, ADAMTS4 (a disintegrin and metalloproteinase with thrombospondin motifs)^7^, tissue inhibitors of metalloproteinases (TIMPs)^8^, interlukine-6 and 8^9^, and even caveolin-1^10^.

Although the etiology of OA is complex, recent evidence has made it apparent that epigenetic changes, altered expression of regulatory RNA and its consequent in gene expression modifications could also participate in the pathogenesis of OA^11^,^12^. MicroRNA (miRNA) is small noncoding RNA with the length of about 20–25 nucleotides (nt), and is transcribed in the nucleus by RNA polymerase II or III. miRNA is involved in regulation of post-transcriptional gene expression by translational suppression or direct degradation of the mRNA via targeting of the coding genes through complementary base pairing between the miRNA and the 3′-Untranslated region (UTR) of the messenger RNA (mRNA) target^13^.

Increasing investigations are evaluating the differential expression of miRNA in OA vs a normal condition. Early study has compared the miRNA profiling between OA patient-derived osteoarthritic cartilage and normal cartilage, and 16 microRNAs have been characterized as osteoarthritis gene signature^14^. Jones *et al.* have identified 17 differential expression miRNAs with more than 4-fold in OA cartilage, and 30 differential expression mRNAs with more than 4-fold in OA bone^14^. Further study has found 12 overexpressed miRNAs in the plasma of patients with primary OA by detecting the expression of 380 miRNAs in OA^15^. Subsequently, some specific miRNAs have shown important roles in OA; for example, miR-140 regulates ADAMTs-5 expression^16^, miR-27b regulates MMP13 expression^17^, and miR-146 is intensely expressed in low grade OA cartilage, and its expression is induced by stimulation of IL-1β, suggesting its involvement in OA pathogenesis^18^. These investigations are highlighting the importance of miRNA in the initiation and development of OA. However, the specific miRNA that regulates expression of OPN in OA is largely unknown. In this study, we have investigated the miRNA that targets expression of OPN.

## Results

### Identification of miRNAs targeting OPN

**Figure 1:**
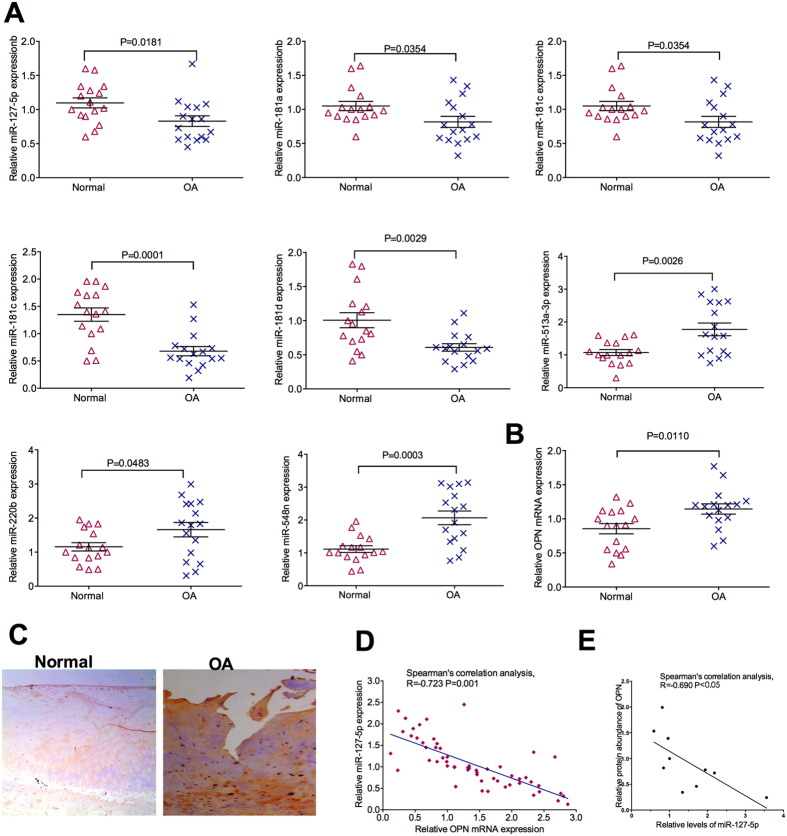
Identification of miRNAs targeting OPN. (**A)** Expression of miR-220b, miR-513a-3p, miR-548n, miR-181a, miR-181b, miR-181c, miR-181d and miR-127-5p in OA patients (n = 16) and the non-OA patients (n = 16). (**B)** Expression of OPN in OA patients (n = 16) and non-OA patients (n = 16). (**C)** Expression of OPN proteins were analyzed using immunohistochemical analyses. Representative images from at least 10 patients for each group. (**D)** The correlation between the expression of miR-127-5p and mRNA expression of OPN. (**E**) The correlation between the expression of miR-127-5p and protein abundance of OPN. OA: osteoarthritis; OPN: osteopontin.

In total, eight potential regulatory miRNAs, including miR-220b, miR-513a-3p, miR-181a, miR-181b, miR-181c, miR-181d, miR-548n and miR-127-5p, were identified by the five algorithms. Next, expression of these miRNAs in OA patients and control were analyzed by RT-PCR. OA patients have higher expression of miR-220b, miR-513a-3p and miR-548n, but lower expression of miR-181a, miR-181b, miR-181c, miR-181d and miR-127-5p, compared to non-OA patients (Fig. 1A). Similar to previous study^4^, mRNA expression of OPN increased in OA patients compared to non-OA patients (Fig. 1B). Also, with analysis from immunohistochemistry, the protein abundance of OPN was higher in OA patients compared to non-OA patients (Fig. 1C). Notably, spearman’s correlation analysis shown that there was a significant negative correlation between expression of miR-127-5p and mRNA expression of OPN (r = −0.723, *P* = 0.001) (Fig. 1D). Similarly, spearman’s correlation analysis shown that there was a significant negative correlation between expression of miR-127-5p and protein abundance of OPN (r = −0.69, P < 0.05) (Fig. 1E). Summarily, OA patients have alterations in expression of miRNAs and OPN, and miR-127-5p may inhibit expression of OPN.

### miR-127-5p inhibits OPN expression

**Figure 2:**
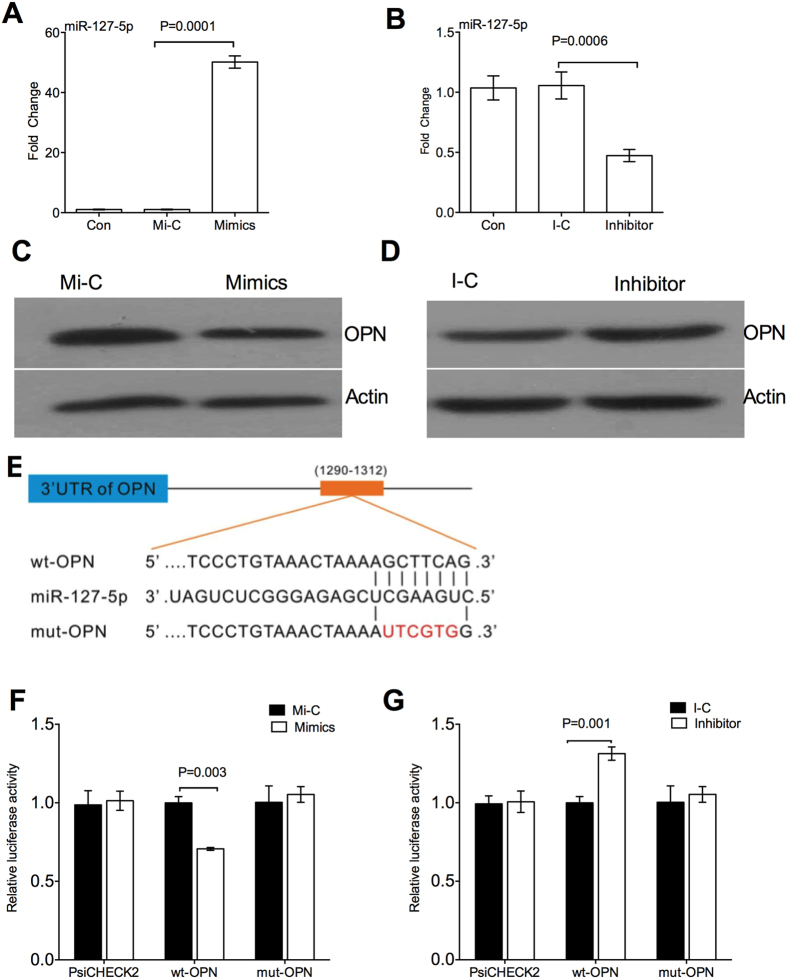
miR-127-5p inhibits OPN expression. (**A,B)** Expression of miR-127-5p in chondrocytes after miR-127-5p mimics (**A**) or miR-127-5p inhibitor (**B**) transfection. **(C,D)** Protein abundance of OPN in the chondrocytes after miR-127-5p mimics (**C**) or miR-127-5p inhibitor (**D**) transfection. (**E**) The illustration of OPN wild- type/mutant 3′-UTRs containing the putative miR-127-5p binding sites. (**F)** The luciferase activity in chondrocytes co-transfected with the wild-type or mutant 3′-UTR reporter vector and the miR-127-5p mimics. (**G)** The luciferase activity in chondrocytes co-transfected with the wild-type or mutant 3′-UTR reporter vector and the miR-127-5p inhibitor. Data are representative of three independent experiments with 4-6 repeats in each time. OPN: osteopontin. Mi-C: miR-127-5p mimics control; I-C: miR-127-5p inhibitor control.

We tested the hypothesis that miR-127-5p directly influences OPN expression by transfecting a miR-127-5p mimics or inhibitor into the chondrocytes and then detecting protein abundance of OPN. miR-127-5p mimics transfection significantly promoted expression of miR-127-5p compared the controls (Fig. 2A), while miR-127-5p inhibitor inhibited expression of miR-127-5p (Fig. 2B). miR-127-5p mimics significantly reduced protein abundance of OPN in the chondrocytes (Fig. 2C), while miR-127-5p inhibitor increased protein abundance of OPN (Fig. 2D), compared to the controls. To directly test the hypothesis that OPN is a downstream target of miR-127-5p, OPN wild-type/mutant 3′-UTRs containing the putative miR-127-5p binding sites were cloned into the psi-CHECK2 reporter vector downstream of the *Photinus pyralis/Renila reniformis* dual luciferase reporter gene (Fig. 2E). Chondrocytes co-transfected with the wild-type 3′-UTR reporter vector and the miR-127-5p mimics showed a significant reduction in luciferase activity, whereas the luciferase activity in the cells transfected with the mutant-type 3′-UTR vector was unaffected by the miR-127-5p mimics (Fig. 2F). We co-transfected the miR-127-5p inhibitor and wild-type 3′-UTR reporter vector into chondrocytes, which demonstrated the luciferase activity was significantly increased in the presence of the miR-127-5p inhibitor (Fig. 2G). Taken together, miR-127-5p inhibits expression of OPN, and OPN mRNA is a downstream target of miR-127-5p.

### miR-127-5p inhibits proliferation of chondrocytes though OPN

**Figure 3:**
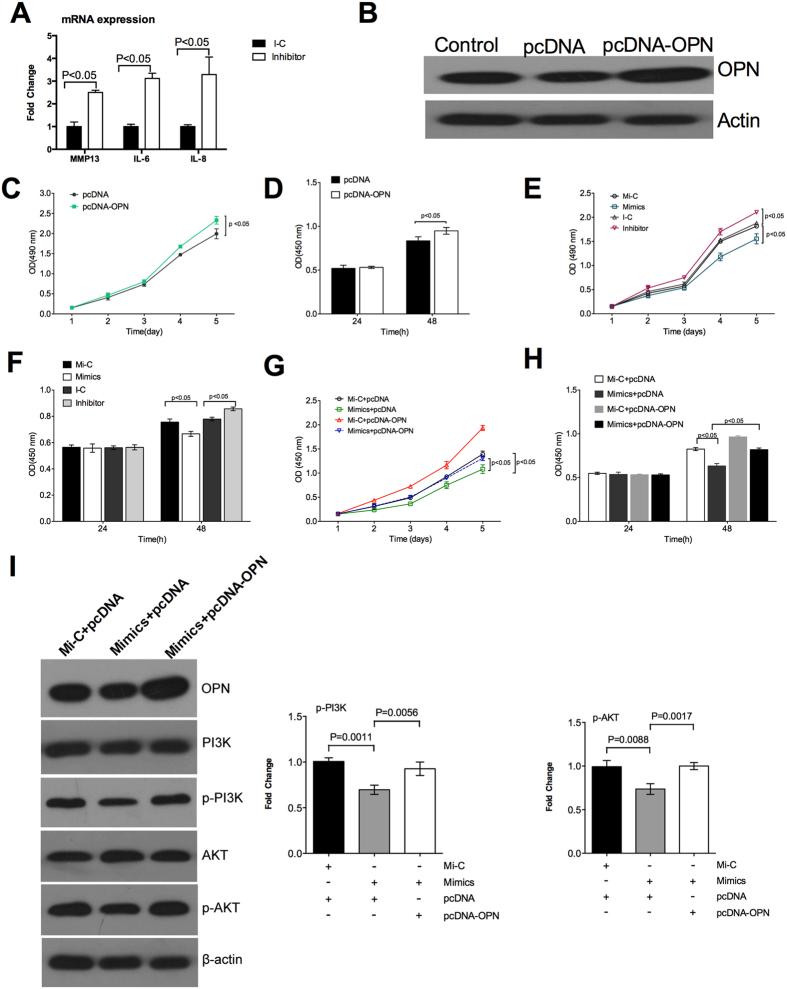
miR-127-5p inhibits proliferation of chondrocytes though OPN. (**A)** mRNA expression of MMP13, IL-6 and IL-8 in the chondrocytes after miR-127-5p inhibitor treatment. B. Protein abundance of OPN in the chondrocytes after pcDNA-OPN transfection. (**C,D)** Proliferation of chondrocytes after pcDNA-OPN transfection by MTT assay (**C**) and BrdU incorporation assays (**D**). (**E,F)** Proliferation of chondrocytes after indicted treatments by MTT assay (**E**) and BrdU incorporation assays (**F**). (**G,H)** Proliferation of chondrocytes after indicted treatments by MTT assay (**G**) and BrdU incorporation assays (**H**). (**I)** The activation of PI3K-Akt pathway by immunoblotting analysis. Data are representative of three independent experiments with 4–6 repeats in each time. OPN: osteopontin; PI3K: phosphatidylinositide 3-kinases; Mi-C: miR-127-5p mimics control; I-C: miR-127-5p inhibitor control.

OPN promotes expression of various factors associated with the pathogenesis of OA, such as MMP13^5^, interlukine-6 and 8^9^. The miR-127-5p inhibitor significantly promoted the mRNA expression of MMP13, IL-6 and IL-8 in the chondrocytes (Fig. 3A). OPN has critical importance on the pathogenesis of OA, associating with the promotion in proliferation of chondrocytes (Fig. 3B–D). miR-127-5p mimics significantly reduced proliferation of chondrocytes, while miR-127-5p inhibitor promoted proliferation of chondrocytes (Fig. 3E,F). To further confirm that OPN is a functional target of miR-127-5p, we rescued the expression of OPN by the transfecting the pcDNA3.1-OPN in chondrocytes, which increased protein abundance of OPN in chondrocytes (Fig. 3B). Although miR-127-5p mimics significantly decreased proliferation of chondrocytes, pcDNA3.1-OPN rescued proliferation of chondrocytes (Fig. 3G,H). Furthermore, although miR-127-5p mimics significantly inhibited the activation of phosphatidylinositide 3-kinase (PI3K)-Akt pathway, pcDNA3.1-OPN rescued the activation of PI3K-Akt pathway (Fig. 3I). Collectively, miR-127-5p inhibits proliferation of chondrocytes by targeting expression of OPN.

## Discussion

Many miRNAs are differentially expressed during OA^14^,^19^,^20^,^21^, including miR-9, miR-98, miR-146a, miR-483, miR-149, miR-582, miR-1227, miR-634, miR-576, miR-641, miR-27a and b, and miRNA-140. However, as far as we known, no study has reported the miRNA that regulates the expression of OPN in OA. OPN is known as early T cell activation gene-1 (Eta-1)^22^,^23^. OPN is secreted by many types of cells, including macrophages, lymphocytes, epithelial cells, vascular smooth muscle cells, and even chondrocytes as well as synoviocytes^24^,^25^,^26^,^27^. OPN is highly abundant in the extracellular fluids at sites of inflammation, extracellular matrix (ECM) of mineralised tissues and even in the bone^24^,^26^,^28^. In the bone, OPN regulates the interactions of cell-matrix and cell-cell, the transitions of cartilage-to-bone in fracture repair, the attachment of osteoclasts to the bone matrix^23^,^29^,^30^. Interestingly, mRNA expression and protein abundance of OPN are associated with the pathogenesis of OA. At the begin, a study found that mRNA expression of OPN isolated from human OA cartilage is higher than the normal cartilage^31^. Subsequently, increased abundance of OPN in the plasma, synovial fluid and articular cartilage in OA patients are found^3^,^32^,^33^, indicating expression of OPN is associated with progressive joint damage, and the severity and progression of OA.

In human breast cancer cell lines (MCF7, MCF10AT and MCF10DCIS.com), hsa-miR-299-5p has been reported to target OPN and regulate the expression of OPN^34^. miRNA 181a targets OPN and decreases OPN expression in hepatocellular cancer cell lines (Hep 3B and Hep G2)^35^, vascular smooth muscle cells^36^. Besides miRNA 181a, miR-220b, miR-513a-3p, miR-181b, miR-181c, miR-181d, miR-548n and miR-127-5p, are also predicted to target and regulate the expression of OPN. Further analysis found that expression of miR-220b, miR-513a-3p and miR-548n increase in OA patients compared to non-OA patients. miR-220b inhibits the autoimmune regulator (AIRE) gene translation through the 3′UTR region of AIRE gene, which is responsible for autoimmune polyendocrinopathy-candidiasis-ectodermal dystrophy^37^. miR-513a-3p has been reported to regulate expression of the luteinizing hormone/chorionic gonadotropin receptor (LHCGR), which is essential for normal male and female reproductive processes^38^. miRNA-548n regulates host antiviral response by direct targeting of Interferon (IFN)-λ1^39^. Although the exact role of miR-220b, miR-513a-3p and miR-548n in the pathogenesis of OA is unknown, it is interesting to investigate the function of increased expression of miR-220b, miR-513a-3p and miR-548n in the establishment and development of OA.

As OPN increased in the pathogenesis of OA^32^, we focused on the down-expressed miRNA, including miR-181a, miR-181b, miR-181c, miR-181d and miR-127-5p. miR-miR181 family members have been reported to regulate the differentiation stages of chondrocyte and chondrocyte formation^40^. Hypertrophic mesenchymal stromal cells (MSC)-derived chondrocytes and non-hypertrophic articular chondrocytes show differential expression of miR-181a^40^. This compelling study is indicating miR181 family members have critical importance in the establishment and development of OA. Indeed, previous reports have shown miR181a directly target and regulate expression of OPN in hepatocellular cancer cell lines (Hep 3B and Hep G2)^35^, vascular smooth muscle cells^36^. Thus, this study mainly focused on the miR-127-5p. miR-127-5p targets the 3′UTR of β-F1-ATPase mRNA (β-mRNA), which is a catalytic subunit of mitochondrial H(+)-ATP synthase and functions in the provision of metabolic energy by oxidative phosphorylation^41^. Notably, miR-127-5p is an important regulator of MMP-13 in human chondrocytes and contributes to the development of OA^42^. This study also shows that miR-127-5p is down regulated in the OA patients, and miR-127-5p targets the 3′UTR of OPN mRNA to down-regulate the expression of OPN. More importantly, we have shown miR-127-5p regulates proliferation of chondrocytes through targeting expression of OPN. Thus, it is fruitful to use miR-127-5p to manipulate the establishment and development of OA.

In conclusion, this study identified that miR-127-5p targets the 3′UTR of OPN mRNA to down-regulate the expression of OPN. In OA, the down-expressed miR-127-5p allows the expression of OPN, which mediates the establishment and development of OA. As far as we known, this is the first study show miR-127-5p directly targets OPN to regulate expression of OPN in OA.

## Materials and Methods

### Cartilage acquisition and assessment

The study was approved by the institutional review board and ethics committee of Xiangya Hospital affiliated to Central South University, which conformed with the regulations of medical ethics. All experiments were conducted in accordance with the approved guidelines. The normal cartilage tissues from non-OA patients and degenerated cartilage tissues from OA patients were obtained in previous study^5^,^6^,^7^,^32^. The cartilage tissues were assessed with hematoxylin- eosin (HE) and safranin-O staining, and a modified Mankin grading system in previous study (Supplementary Figure 1)^5^,^6^,^7^,^32^. A written informed consent about this experiment was obtained from all subjects.

### Cell isolation and culture conditions

The chondrocytes were isolated and cultured according to previous study^5^,^6^,^7^. Briefly, samples were minced into pieces of less than 1 mm^3^, followed by sequential digestion at 37 °C with 0.15% collagenase II (Invitrogen, Carlsbad, CA, USA) for 5–6 h with stirring every 20 min after 2 h. Chondrocytes were isolated after centrifugation and cultured in DMEM-F12 containing 10% fetal bovine serum (FBS) and antibiotics for 5–7 days before use.

### miRNA prediction

To predict the miRNA targeting in the 3′UTR of OPN, five miRNA prediction programs, RNA22, TargetScan, miRDB, miRWalk and miRanda, were used to confirm the same target binding sites.

### Recombinant plasmid construction

The 3′-UTR sequence of OPN (TCC CTG TAA ACT AAA AGC TTC AG) containing the putative miR-127-5p binding site were synthesized by Invitrogen (USA). The mutant sequence (TCC CTG TAA ACT AAA AUT CGT GG) by mutating the seed regions of the miR-193b binding sites was also synthesized by Invitrogen (USA). The synthesized products were cloned into the psiCHECK-2 vector (Invitrogen, USA). The recombinant plasmids were named as psiCHECK-2-OPN-wt and psiCHECK-2-OPN-mut. OPN expression vector was established for the “rescue” experiment, where the open reading frame of OPN was cloned into pcDNA3.1 (Invitrogen, USA). The recombinant plasmid was named as pcDNA3.1-OPN.

### Cell transfection

The miR-127-5p mimics, inhibitors and their negative controls (NC) were purchased from Promega (USA). Chondrocytes were transfected with pcDNA3.1 (2 *μ*g/mL), psiCHECK-2 reporter plasmid (2 μg/mL), miR-222 mimics (50 nM), inhibitor (50 nM) or their negative controls (50 nM) using Lipofectamine 2000 (Invitrogen, USA) according to the manufacturer’s instructions.

### Luciferase assay

Chondrocytes were transfected with psiCHECK-2-OPN-wt, psiCHECK-2-OPN-mut, miR-127-5p mimics, miR-127-5p inhibitor or its control using Lipofectamine 2000 reagent. After 48 h transfection, cells were lysed, and assays were performed using the Dual-Luciferase Reporter Assay System kit (Promega, USA) according to the manufacturer’s instructions.

### BrdU incorporation assays

Chondrocytes were cultured for 16 h, and then pulsed with 5-Bromo-2-deoxyuridine (BrdU) for an additional 8 h. Cell proliferation was determined by BrdU incorporation assay according to the manufacturer’s instructions (Roche Diagnostics GmbH, Roche Applied Science, Germany).

### MTT assay

Cell viability was assayed by using 3-(4,5)-dimethylthiahiazo(-z-y1)-3,5-di-phenytetrazoliumromide (MTT). After treatment, 10 μL MTT (5 mg/mL) was added into cultured medium in each well for 2–4 hours until purple precipitate is visible. After the removal of culture medium, 75 μL dimethyl sulphoxide was added to each well, leaving the cells at room temperature in the dark for 2 hours. The absorbance at 570 nm was recorded.

### RT-PCR

RT-PCR analysis was performed according to previous reports^43^,^44^,^45^. Briefly, total RNA was isolated from liquid nitrogen frozen samples using TRIZOL regent (Invitrogen, USA) and then treated with DNase I (Invitrogen, USA) according to the manufacturer’s instructions. Synthesis of the first strand (cDNA) was performed using oligo (dT) 20 and Superscript II reverse transcriptase (Invitrogen, USA). Primers used in this study were designed with Primer 5.0. Sequences of all primers used were: OPN-F: 5′-GTGGGA AGG ACA GTT ATG AA-3′; OPN-R: 5′-CTG ACT TTG GAA AGT TCC TG-3′; GAPDH-F: 5′-TGA CTT CAA CAG CGA CAC CCA-3′; GAPDH-R: 5′-CAC CCT GTT GCT GTA GCC AAA-3′; MMP13-F: 5′-CTT AGA GGT GAC TGG CAA AC-3′; MMP13-R: 5′- GCC CAT CAA ATG GGT AGA AG -3′. The primer pair for IL-6 was made to bp 42–61 (sense) and bp 334–354 (antisense) according to IL-6 cDNA sequence, and the IL-8 primer pair was made to the bp 147–174 (sense) and bp 342–366 (antisense). GAPDH was used as an internal control to normalize target gene transcript levels.

Expression of mature miRNA was quantified using a TaqMan miRNA assay kit (Applied Biosystems). Purified miRNA was reverse transcribed using a TaqMan miRNA RT kit (Applied Biosystems) and miRNA-specific stem-loop RT primers (Applied Biosystems). Real-time PCR was performed using a StepOnePlus Real-time PCR System (Applied Biosystems) in a 10 μL PCR mixture containing 2 μL RT product, 5 μLTaqMan Universal PCR Master Mix, 0.2 μM TaqMan probe, and 10 μM forward and reverse primers. RNU6B was used as an internal control for miRNA detection.

### Immunoblotting

Western blot analysis was conducted according to previous study^46^,^47^,^48^. Equal amounts of proteins obtained from samples were separated by SDS-PAGE, transferred to PVDF membranes (Millipore, MA, USA), and blocked with 5% non-fat milk in Tris-Tween buffered saline buffer (20 mM Tris, pH 7.5, 150 mM NaCl, 0.1% Tween-20) for 3 h. Antibodies against OPN (AP11567a, Abgent, CA, USA), PI3K (ab86714, Abcam, MA, USA), p- PI3K (ab182651, Abcam, MA, USA), Akt (ab8805, Abcam, MA, USA) or p-Akt (ab38449, Abcam, MA, USA) were incubated overnight at 4 °C and HRP-conjugated secondary antibodies were incubated for 1 h at room temperature before development and analysis using Alpha Imager 2200 software (Alpha Innotech Corporation, CA, USA). Signal intensity was digitally quantified and normalized to actin protein abundance.

### Statistical analyses

Data shown are the means ± the standard error of the mean (SEM). All statistical analyses for data were performed using SPSS 16.0 software (Chicago, IL, USA). Data were analyzed between two groups using the Student’s t-test, while among more than two groups by the One-Way ANOVA method^48^,^49^,^50^,^51^. Differences of p < 0.05 were considered significant.

## Additional Information

**How to cite this article**: Tu, M. *et al.* MicroRNA-127-5p regulates osteopontin expression and osteopontin-mediated proliferation of human chondrocytes. *Sci. Rep.* **6**, 25032; doi: 10.1038/srep25032 (2016).

## Supplementary Material

Supplementary Information
