# Supplementary material for: MicroRNA-127-5p regulates osteopontin expression and osteopontin-mediated proliferation of human chondrocytes
Source: Sci Rep. 2016 Apr 29;6:25032. doi: 10.1038/srep25032 (PMC4850404; doi:10.1038/srep25032)
Supplement: Supplementary Information [file srep25032-s1.doc]

**MicroRNA-127-5p regulates osteopontin expression and osteopontin-mediated proliferation of human chondrocytes**

Min Tu,Yusheng Li, Chao Zeng, Zhenhan Deng, Shuguang Gao,Wenfeng Xiao, Wei Luo,Wei Jiang, Liangjun Li, Guanghua Lei

Supplementary figure 1. Hematoxylin-eosin staining, safranin-O staining and modified Mankin score. A. With hematoxylin-eosin staining (×100 magnification), OA degenerative cartilage shows an irregular exfoliative surface of cartilage, and chondrocytes cluster in disorganized cartilage lacuna, while normal cartilage shows a much more regular surface of cartilage, and chondrocytes distribute neatly and evenly in the cartilage lacuna. B. With Safranin-o staining (×100 magnification), OA degenerative cartilage is deep stained or stained loss, while normal cartilage is natural and tinged. C. Comparison of modified Mankin score (mean ± SD) between OA cartilage and normal cartilage. OA: osteoarthritis


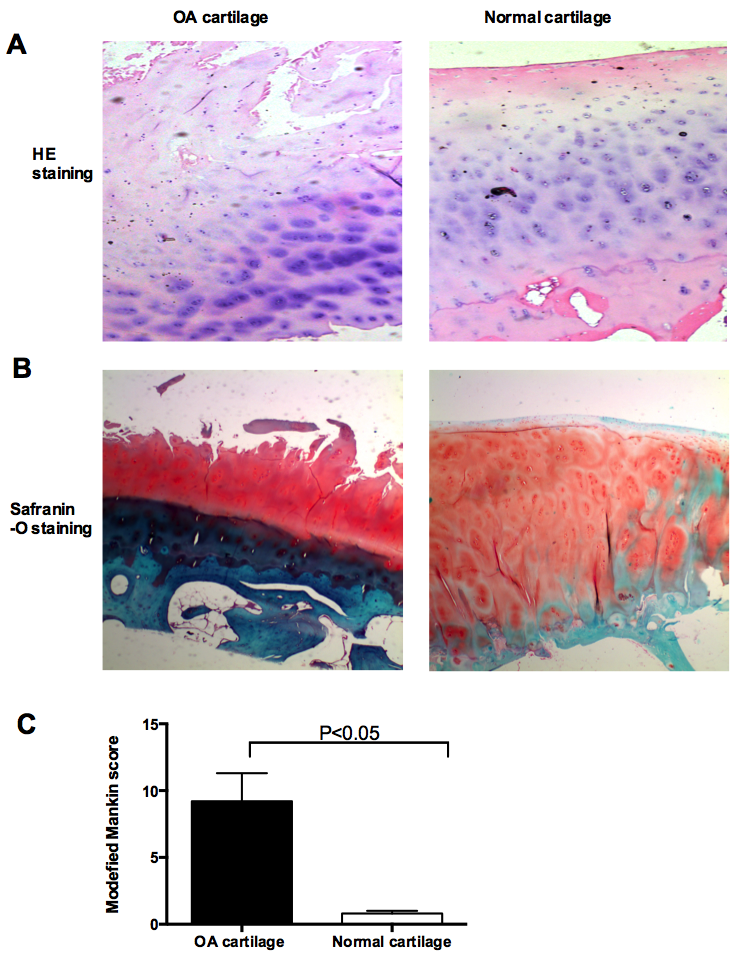


Supplementary figure 1
